# Prevalence and Impact of Revenge Pornography on a Sample of Portuguese Women

**DOI:** 10.1007/s12119-023-10100-3

**Published:** 2023-06-03

**Authors:** Ana Murça, Olga Cunha, Telma Catarina Almeida

**Affiliations:** 1 Egas Moniz School of Health & Science, 2829-511 Caparica, Almada Portugal; 2 Universidade Lusófona - Centro Universitário do Porto, Hei-Lab: Digital Human-Environment Interaction Labs, 4000-098 Porto, Portugal; 3 Egas Moniz Center for Interdisciplinary Research (CiiEM), Egas Moniz School of Health & Science, 2829-511 Caparica, Almada Portugal; 4 LabPSI – Laboratório de Psicologia Egas Moniz, Egas Moniz School of Health & Science, Caparica, Almada Portugal

**Keywords:** Revenge pornography, Internet, Victims, Impact, Humiliation

## Abstract

Victims of revenge pornography (RP) suffer long-term psychological, personal, and social consequences, given that the spread of explicit content may continue to disturb them throughout their lives. However, there is a scarcity of studies on this phenomenon in Portugal. The present study aims to identify the prevalence of RP and analyze its impact on self-esteem, humiliation, depression, and anxiety, and compare victims and non-victims of RP on these same variables. The sample comprises 274 Portuguese women aged between 18 and 82. The data was collected through an online protocol consisting of a sociodemographic questionnaire, the Rosenberg Self-Esteem Scale, the Humiliation Inventory, and the Brief Symptoms Inventory. From the total sample, 45 (16.4%) participants reported at least one experience of RP. RP victims reported higher levels of humiliation, anxiety, and depression and lower levels of self-esteem than non-victims. However, only humiliation distinguished RP victims and non-victims. RP is a growing phenomenon enhanced by the intensified use of technology. Along with this phenomenon comes the impact on victims, which has long-term consequences. This study contributes to the scientific community since the scientific study of RP and its impact on victims is still incipient.

## Introduction

Technology facilitates new means of communication, and the available technological tools seem to provide greater digital space for victimization and for the offender to practice harmful actions (Stonard, 2020). Furthermore, the COVID-19 pandemic intensified social isolation, resulting in the more frequent use of technological tools (Adibelli & Sumen, 2020). This situation reinforced the practice of revenge pornography (RP) through the Internet, causing adverse damages to the victims' lives (Souza, 2020).

The definition of RP remains controversial in the literature. Some authors define RP as the perpetration of certain behaviors, such as sending text messages and explicit images (Cooper et al., 2016; Diliberto & Mattey, 2009; Drouin et al., 2013), while others (e.g., Döring, 2014) define this phenomenon exclusively through the sharing of sexual images. Despite the controversies, the most robust definition conceptualizes RP as a practice that occurs when a person, usually an ex-partner, shares sexually explicit content (e.g., videos, photographs) without the victim's consent, for retaliation, frequently after the end of an intimate relationship (Bloom, 2014). However, the perpetrator may be an acquaintance of the victim, such as a co-worker, a friend, an ex-boyfriend, or even a cyberstalker who has gained access to the intimate content of the victim (Rallan & Vig, 2019).

Sexting and RP are two related constructs. However, while RP is based on sharing explicit content for retaliation, sexting is the consensual exchange of sexually explicit material through text messages, images, videos, smartphones, the internet, or social media, without the purpose of causing harm (Chalfen, 2009). The RP phenomenon typically involves an ex-partner sending images with explicit content (nude or semi-nude) obtained through sexting (Bates, 2017). There is paucity on the literature on RP in Portugal. Therefore, the present paper aims to identify the prevalence of RP among Portuguese women and analyze the impact of RP on victims' self-esteem, humiliation, and psychological symptoms.

### The Use of the Internet as a Facilitator for Revenge Pornography

In the United States, the Pew Research Center (2018) study shows that 98% of young people between the ages of 18 and 29 use the Internet, and 86% of college-age adults reported that, while on social networks, such as Facebook or Twitter, they share personal information (Varghese & Pistole 2017). Pitcher's (2016) study indicated that nearly 50% of people who own cell phones had used them to take some form of an intimate image.

The Data and Society Research Institute (2016) reported that approximately one in 25 Americans are either threatened by or are victims of non-consensual image sharing, which equates to approximately 10 million Americans. Another study showed that one in five people had been a victim of RP, with 16 and 29 years old as the most prevalent age group (Powell et al., 2017). Although the perpetrator can be anyone, the study by Silva and Souza (2020) reported that in 86% of RP cases, the perpetrator, that is, the person who disseminated the victim's images, had a personal relationship with the victim, such as an ex-boyfriend. Victims revealed that contact with the offender could happen in several ways, the most frequently through social media (54%), messaging apps (41%), video-voice call apps (23%), email (12%), dating apps (9%), and gaming programs (4%) (O’Connor et al., 2018). As a result of these incidents, firms like Twitter and Reddit implemented policies prohibiting any form of RP on their platforms (Pollack, 2017), while other sites, such as Facebook, allow a victim to request the removal or restriction of an explicit photograph (Branch et al., 2017).

Stonard (2020) study reported that offenders saw the use of technological media as an easier way to perpetrate abusive behaviors, as they become more confident or more able to act in an abusive or controlling manner from behind a screen, anonymously, instantaneously, and constantly. The use of technological tools, mainly social media, to facilitate sexual violence and harassment is a phenomenon that is becoming increasingly significant in studies of interpersonal violence (Henry & Powell, 2014; Powell & Henry, 2019).

### Revenge Pornography as Gender Violence

There is some controversy in the literature regarding the position of RP as a modality of gender violence. Ringrose et al. (2012) argue that young males often resort to threats and image sharing to expose, humiliate, or spread rumors about a female partner. Socially and culturally normalized sexism legitimizes and reinforces these behaviors, meaning that these events are not gender neutral. Cavalcante and Lelis (2016) reinforced this idea, saying that RP practices are mostly against women, thus denoting a new modality of aggression that characterizes gender violence. Souza (2020) added that RP primarily targets female victims, about 90%, which denotes a gender problem, culturally constructed in society over the decades (Cavalcante & Lelis, 2016).

On the other hand, Walker and Sleath 2017) argued that rates of non-consensual sharing and victimization fluctuate significantly depending on the definition and operationalization of the behavior, and the RP behaviors are evident for a considerable quantity of individuals of both genders. Powell et al.’ (2017) study reinforces the previous perspective that both men (23%) and women (22%) are equally likely to report being victims of RP, revealing that this type of abuse is not exclusively a form of gender violence, according to these authors' perspective.

### The Impact of Revenge Pornography

RP has adverse mental health consequences for victims. Several studies (e.g., Aborisade, 2022; Bates, 2017; Campbell et al., 2022; Citron & Franks, 2014; Kamal & Newman, 2016; Rallan & Vig, 2019) noted that victims of RP report public shame, humiliation, powerlessness, problems in intimate relationships, making it difficult to start new relationships, mental health effects (e.g., suicidal thoughts, anxiety) (Mckinlay & Lavis 2020), job loss and/or difficulty in finding a new job (Campbell et al., 2022). Kamal and Newman (2016) stated that victims of RP suffer a long-term psychological, personal, and social negative impact, given that the explicit content disseminated may continue to disturb them throughout their lives. RP victims are also more susceptible to new forms of victimization, such as harassment and stalking (Mckinlay & Lavis 2020).

Over the years, high levels of self-esteem have been identified in the literature as one of the characteristics most associated with happier individuals (Myers and Diener 1995), in contrast to what has been seen with victims of RP (Bates, 2017). The few studies in the literature focusing on this phenomenon reveal that RP victims often report low self-esteem levels (Aborisade, 2022; Bates, 2017). RP victims also tend to feel humiliated (Bond & Tyrrell, 2018; Citron & Franks, 2014) and experience trust issues (Bates, 2017). Humiliation is an outcome of interpersonal or social interactions which diminish or even ruin an individual's psychological well-being and fundamental sense of dignity (worth) by disrupting affective-emotional organization (Hartling & Lindner, 2016; Leidner et al., 2012).

According to the literature, there is also a link between mental health problems (e.g., depression and anxiety) and online victimization, such as RP (e.g., Klettke et al., 2014). Posttraumatic stress disorder, depression, anxiety, and suicidal thoughts are identified as adverse outcomes of RP (Aborisade, 2022; Bates, 2017; Campbell et al., 2022; Priebe & Svedin, 2012). Victims of RP reported high levels of psychological distress, consistent with a diagnosis of moderate to severe depression and/or anxiety disorder, being almost twice as likely as non-victims to report experiencing psychological distress (Henry et al., 2017). Some studies suggested that the negative health consequences of RP for female victims are similar to those found among rape victims (Bates, 2017) or child pornography victims (Kamal & Newman, 2016).

### Present Study

The study of RP in an international context is yet incipient. There is a notable lack of studies on this type of violence in Portugal. However, Portugal adopted the definition as the non-consensual sharing of intimate images by one partner to the other, usually at the end of a relationship (Associação Portuguesa de Apoio à Vítima [APAV; Portuguese Association for Victim Support], 2020). The sharing of intimate content between partners and its subsequent dissemination on the internet is conducted through various digital tools. We can consider that RP is a current issue highly associated with the increasing use of technology and violence in intimate relationships. The increasing use of technological tools (e.g., social networks), easy access to the internet, and its use to perpetrate violence against others are increasingly present in individuals' daily lives, which brings risks to internet users. Thus, this study aims to contribute to scientific knowledge about a new form of online violence, which seems to grow exponentially, alerting people to the new risks and harmful ways associated with internet use. Therefore, the present research aims to identify the prevalence of RP among Portuguese women and understand such violence's impact on self-esteem, humiliation, anxiety, and depression. More specifically, we aim to: (1) analyze the prevalence of RP among Portuguese women from the community; (2) identify the psychological impact of such type of violence; and (3) compare victims and non-victims of RP concerning self-esteem, humiliation, depression, and anxiety symptoms.

## Method

### Participants

Table 1 presents the sociodemographic characteristics of a convenience sample of 274 Portuguese women aged between 18 and 82 (*M* = 28.08, *SD* = 10.48). Most participants identify as heterosexual (*n* = 223, 81.4%), and 210 (76.6%) are single. Most participants (*n* = 115, 42%) have a college degree, and 51.8% (*n* = 142) of the sample are employed. Finally, most participants were in a romantic relationship (*n* = 169, 61.7%).

**Table 1:** Sociodemographic characterization of the sample (*n* = 274)

	*n*	%
Sexual orientation		
Heterosexual	223	81.4
Non-heterosexual	51	16.4
Marital status		
Single	210	76.6
Married/cohabitation	47	17.2
Separated/divorced	16	5.8
Widower	1	0.4
Education level		
Until 9th grade	8	2.9
12th grade	85	31
Graduation	116	42.3
Master’s degree	65	23.7
Professional status		
Student	113	41.2
Employed	142	51.8
Unemployed	14	5.1
Retired	5	1.8
Intimate relationship		
Yes	169	61.7
No	105	38.3

*n* number of participants; % percentage of participants

### Measures

#### Sociodemographic Questionnaire

The sociodemographic questionnaire was applied to collect the following variables: age, sex, nationality, sexual orientation, marital status, academic qualifications, employment status, profession, and whether they are currently in a romantic relationship.

#### Revenge Pornography Victimization Checklist

The checklist was created for this study and was based on items from other questionnaires, which include questions such as “Some of your partners have taken pictures or videos of you with sexual or suggestive content (e.g., on the beach, in a dress) without your consent and then disseminated the content via cell phone or the Internet” and “Some of your partners have posted online sexually suggestive messages or images (of you) on your profile that you did not want”. The RP victimization checklist presents six dichotomous items (Yes/No) to assess the presence of RP-related victimization behaviors. The six items are then summed to create a dichotomous variable (Yes/No): victim of RP if participants reported at least one RP behavior, and non-victim of RP, if participants did not report any RP behavior.

#### Rosenberg Self-Esteem Scale (RSES; Rosenberg, 1965; Portuguese version Pechorro et al., 2011)

RSES aims to assess self-esteem in adolescents and adults. It is a brief self-report measure with 10 items, consisting of a Likert-type scale of 4 points (0 – Strongly Disagree; 3 – Strongly Agree), with scores ranging from 0 to 30. Higher scores indicate higher levels of self-esteem. The Portuguese version of the scale presents a good internal consistency (*α* = 0.81). In our study, the scale has a good Cronbach's alpha (*α* = 0.92).

#### Humiliation Inventory (HI; Hartling & Luchetta, 1999; Portuguese version Cardoso et al., 2019)

HI aims to assess individuals' feelings of humiliation towards an event. The inventory consists of 32 items, rated on a 5-interval Likert scale (1—Not at all; 5—Very Much), with scores ranging from 32 to 160. High scores correspond to a high degree of experience of humiliation. The reduced Portuguese version of the HI comprises three factors—cumulative humiliation, fear of humiliation, and concern/worry about being a victim of humiliation. The instrument has a good internal consistency for the total scale (*α* = 0.96) in the original study, as it is for the Cumulative Humiliation Subscale (*α* = 0.94) and Fear of Humiliation (*α* = 0.96). The Portuguese version of the scale showed a good internal consistency (*α* = 0.96), as in our study (*α* = 0.97).

#### Brief Symptoms Inventory (BSI; Derogatis, 1982; Portuguese version Canavarro, 2007)

BSI is a self-report measure that consists of 53 items assessing psychological symptoms. Items are rated on a 5-point scale (0—Not at all; 4—Extremely), which reflect the level of distress an individual has experienced on each of the symptoms during the previous seven days. The inventory is composed of nine dimensions—somatization, obsession–compulsion, interpersonal sensitivity, depression, anxiety, hostility, phobic anxiety, paranoid ideation, and psychoticism—and three global indices of distress—Global Severity Index (GSI), Positive Symptom Distress Index (PSI), and Positive Symptom Total (PST). In the present study, only depression and anxiety dimensions were used. The Portuguese version of the instrument showed adequate internal consistency for the depression (*α* = 0.73) and anxiety (*α* = 0.77) dimensions. The Cronbach's alpha in our study was good for depression (*α* = 0.94) and anxiety (*α* = 0.93).

### Procedure

The sample was collected through an online survey. The protocol was inserted in a Google Forms platform and disseminated by e-mail (e.g., researchers’ contacts, universities/institutional mailing lists) and social networks (e.g., LinkedIn, Facebook) from January to March 2022. Individuals should be Portuguese adult women who can read and write to participate in the study. All participants signed the electronic informed consent form to inform them of the purpose of the study and all procedures involved. All ethical aspects regarding data collection were performed to safeguard the well-being of all participants and the participants' anonymity. This study was performed according to the principles of the Declaration of Helsinki (World Medical Association, 2013). The Egas Moniz School of Health and Science Ethics Committee approved this research.

### Statistical Analysis

The statistical software IBM SPSS, Version 28, was used for data analysis. We used descriptive statistics through central and dispersion tendency measures to describe the participants and the prevalence of RP. T-tests were conducted to compare groups in sociodemographic and psychological variables. Two binary logistic regressions were used to test associations between the predictive variables (sociodemographic and psychological variables—total scores and subscales) and the dependent variable (non-victim of RP vs. victim of RP).

## Results

### Prevalence of Revenge Pornography Victimization

Table 2 shows that from the total sample, 45 (16.4%) women reported being victims of at least one act of RP lifetime. The type of action most suffered was sharing private or embarrassing information about themselves through text messages or social media without permission (*n* = 29, 10.6%), followed by sharing through a mobile phone or internet compromising (of sexual nature, suggestive or insinuating) images or videos of themselves without permission (*n* = 19, 6.9%), sending others photos or private intimate videos who shared with him/her without permission (*n* = 19, 6.9%), and pressing themselves to do things that they do not want (independently if they finally agree or not) threatening themselves with the dissemination of the intimate conversations or images (*n* = 19, 6.9%). The other behaviors were less reported.

**Table 2:** Prevalence of revenge pornography acts (*n* = 274)

	*N*	%
Sharing private or embarrassing information about themselves through text messages or social media without permission	29	10.6
Sharing through cell phone or internet compromising images or videos of themselves (of a suggestive or insinuating sexual nature) without permission	19	6.9
Sending, without permission, to others intimate private photos or videos shared with him/her	19	6.9
Pressing themselves to do things that they do not want (independently if you finally agree or not) threatening themselves with the dissemination of the intimate conversations or images	19	6.9
Taking pictures or recording videos of themselves with a sexual or suggestive content (e.g., in the beach, in a locker room) without their consent and then disseminated that content through cell phone or internet	13	4.7
Posting online messages or sexually explicit images in their online profile without permission	7	2.6

### Impact of Revenge Pornography

Table 3 shows that women victims of RP revealed an average of 16.11 (*SD* = 6.67) in self-esteem, a significantly lower level than those found among the general Portuguese population (Santos & Maia, 2003). Regarding humiliation, women victims of RP showed an average score of 76.27 (*SD* = 20.78). This score, as well as the scores of the subscales, cumulative humiliation (*M* = 38.40, *SD* = 9.11), fear of humiliation (*M* = 22.04, *SD* = 9.47), and concern/worry (*M* = 15.82, *SD* = 5.95) was higher than those found for the general Portuguese population (Cardoso et al., 2019). For anxiety and depression symptoms, women victims presented an average score of 2.68 (*SD* = 1.05) and 2.64 (*SD* = 1.09), respectively. Again, these scores were higher than those for the general Portuguese population (Canavarro, 2007).

**Table 3:** Differences between victims (*n* = 45) and non-victims (*n* = 229)

	Victims		Non-victims		χ^2^	*p*	*Cramer V*
	*n*	%	*n*	%			
Sexual orientation							
Heterosexual	32	71.1	191	83.4	3.753	.053	.117
Non-heterosexual	13	28.9	38	16.6			
Marital status							
Single	41	91.1	169	73.8			
Married/cohabiting	4	8.9	43	18.8	6.955	.073	.159
Separated/divorced	0	0	16	7.0			
Widower	0	0	1	0.4			
Educational level							
Until 9th grade	0	0	8	3.5			
Until 12th grade	21	46.7	64	27.9	7.207	.066	.162
Graduation	16	35.6	100	43.7			
Master’s degree	8	17.8	57	24.9			
Professional status							
Student	23	51.1	90	39.3			
Employed	22	48.9	120	52.4	5.095	.165	.165
Unemployed	0	0	14	6.1			
Retired	0	0	5	2.2			

	*M*	*SD*	*M*	*SD*	*t*	*p*	*d*
Age	24.11	5.36	28.86	11.06	2.815	.005	.459
Total RSES	16.11	6.67	19.72	6.63	3.336	< .001	.544
Total humiliation	76.27	20.78	58.84	23.75	− 4.588	< .001	.748
Cummulative humiliation	38.40	9.11	28.10	11.73	− 5.565	< .001	.907
Fear humiliation	22.04	9.47	17.14	9.40	− 3.199	< .001	.522
Concern/worry	15.82	5.95	13.60	6.52	− 2.120	.017	.346
BSI depression	2.64	1.09	1.97	1.14	− 3.651	< .001	.595
BSI anxiety	2.69	1.05	1.98	1.23	− 3.597	< .001	.587

*RSES* Rosenberg Self-Esteem Scale, *BSI* Brief Symptom Inventory

### Comparison Analyses Between Victims and Non-victims

Regarding sociodemographic variables, comparison analyses only revealed statistically significant differences between RP victims and non-victims on age, *t*(272) = 2.815, *p* = 0.005, *d* = 0.459. Thus, victims of RP tend to be younger than non-victims.

Concerning psychological variables (see Table 2), statistically significant differences were found between victims of RP and non-victims on self-esteem, *t*(272) = 3.336, *p* < 0.001, *d* = 0.544, depression, *t*(272) = − 3.651, *p* < 0.001, *d* = 0.595, anxiety, *t*(272) = − 3.597, *p* < 0.001, *d* = 0.587, total humiliation, *t*(272) = − 4.588, *p* < 0.001, *d* = 0.748, cumulative humiliation, *t*(272) = − 5.565, *p* < 0.001, *d* = 0.907, fear of humiliation, *t*(272) = − 3.199, *p* = 0.002, *d* = 0.522, and concern/worry, *t*(272) = − 2.120, *p* = 0.035, *d* = 0.346. RP victims showed lower self-esteem levels and higher levels of depression, anxiety, total humiliation, cumulative humiliation, fear of humiliation, and concern/worry than non-victims.

### Psychological Variables as a Function of Revenge Pornography Victimization

Two binary logistic regressions were conducted to find which psychological variables are associated with RP victimization (see Table 4). RSES total scores, humiliation total scores and subscales, depression, and anxiety scales are entered as predictors in two independent models after controlling for age.

**Table 4:** Logistic regression coefficients of psychological variables as a function of revenge pornography victimization (*n* = 274)

	Total scores								Subscales scores							
	Model 1				Model 2				Model 1				Model 2			
	B	S.E	*p*	Exp(B)	B	S.E	*p*	Exp(B)	B	S.E	*p*	Exp(B)	B	S.E	*p*	Exp(B)
Age	−.070	.026	.008	.932	−.056	.029	.052	.946	−.070	.026	.008	.932	−.054	.029	.060	.947
RSES					.008	.037	.829	1.008					−.015	.039	.141	.707
Depression					.076	.283	.788	1.079					−.191	.303	.530	.827
Anxiety					.141	.255	.582	1.151					.146	.257	.570	1.157
Humiliation total					.008	.037	.031	1.024								
Cumulative humiliation													.077	.023	< .001	1.081
Fear of humiliation													.020	.031	.506	1.021
Concerns/worry													−.048	.045	.289	.953

*RSES* Rosenberg Self-Esteem Scale

In both models, the variable included in the first step produced a statistically significant model, χ^2^(1) = 10.326, *p* = 0.001. The role of age produced a pseudo-R between 3.7% (Cox and Snell) and 6.3% (Nagelkerke), revealing that the model accurately classified 83.6% of the cases.

When RSES total score, humiliation total score, depression, and anxiety were added to this analysis, the model was statistically significant, χ^2^(4) = 15.992, *p* = 0.003, as well as the final model, χ^2^(5) = 26.318, *p* < 0.001. This set of variables produces a pseudo-R^2^ between 9.2% (Cox & Snell) and 15.5% (Nagelkerke). The model accurately classified 83.6% of the cases. Only the humiliation total score contributed significantly to the model (OR 1.024; 95% CI [1.002; 1.046]). Thus, for each unit increase in humiliation total score, the odds of being a victim of RP increased by a factor of 1.024.

After including humiliation subscales, the full model remained statistically significant, χ^2^(6) = 25.623, *p* < 0.001, as well as the final model, χ^2^(7) = 35.949, *p* < 0.001, accounting for between 12.3% (Cox and Snell) and 20.8% (Nagelkerke) of the variance. The overall classification accuracy rate was 83.6%. Only cumulative humiliation contributed significantly to the model (OR 1.081; 95% CI [1.032; 1.131]). Thus, each unit increase in cumulative humiliation multiplies the odds of being a victim of RP by 1.081.

## Discussion

The present study aims to identify the prevalence of RP among a sample of Portuguese women from the community and analyze the impact of this type of victimization on self-esteem, feelings of humiliation, and symptoms of depression and anxiety, examining differences between victims of RP and non-victims. In Portugal, to date, there are no scientific studies about RP, and there are only few studies that address the thematic at an international level.

The development of technological tools and different digital platforms has enabled an exponential growth of online users over the years, which, in turn, brings risks associated with internet use. The lack of information and inappropriate use of technological tools can cause severe damage to the victim's mental health, often negatively impacting their daily routines. This research revealed that 16.4% of the total sample had experienced at least one act of RP throughout their lives. The most reported act by victims is sharing private information without their permission. Other studies support the present results, revealing that one in five people have seen their intimate content disseminated online without permission (e.g., Powell et al., 2017). The age range of victims in our study is also similar to those found in other studies, i.e., young adults (Powell et al., 2017). Our study shows that victims of RP tend to be younger than non-victims. This result might be explained by the high rates of Internet use (Pew Research Center, 2018) and the high sharing of personal information, including nudes and other intimate content, through social networks among young people (Varghese & Pistole, 2017).

Our study also revealed that private information is spread most frequently through social media or text messages. These results are aligned with other research, showing that contact with offenders is established primarily through social media and messaging apps, resulting in RP (e.g., O’Connor et al., 2018). This study revealed that RP victims have higher mental health problems, such as depression and anxiety, than non-victims. The presence of depression, anxiety, and suicidal ideation are some of the consequences of RP on victims, also identified in other studies (Aborisade, 2022; Bates, 2017; Campbell et al., 2022). Those victims find it more difficult to socialize with others, leading to social isolation because of their experience of anxiety and depression (Campbell et al., 2022). Other studies have already identified the social costs of RP on victims (e.g., Branch et al., 2017). High scores of psychological and emotional reactions in victims of RP may be a consequence of the protracted psychological trauma they are subjected to when facing the sexual images that were shared (Aborisade, 2022).

Furthermore, victims of RP tend to feel a deep sense of betrayal, powerlessness, worthlessness, and low self-esteem (Aborisade, 2022). In the current study, victims of RP also showed lower self-esteem than non-victims, in line with other studies (e.g., Aborisade, 2022; Priebe & Svedin, 2012). Victims tend to self-blame for their victimization (Aborisade, 2022), which negatively affects their psychological well-being and emotional balance (Baumeister et al., 2003), leading to the development of lower levels of self-esteem. The positive concepts (e.g., well-being, self-efficacy) associated with self-esteem tend not to be present in victims of RP, as they suffer some form of power and pressure from the perpetrator on them at the time of sharing the content, which tends to destroy employment opportunities, effect job loss, family, and social relationships, leading to lower levels of self-esteem (Bates, 2017). This type of victimization can be a traumatic experience because victims perceive negative social attitudes toward them (Aborisade, 2022).

Another pertinent finding of this study was the high levels of humiliation feelings among victims of RP and the significantly higher scores compared with non-victims. When all the variables were considered, only humiliation revealed significant results, showing that cumulative humiliation increases the probability of belonging to the group of RP victims. Other research (e.g., Bond & Tyrrell, 2018; Citron & Franks, 2014; Rallan & Vig, 2019) also reveals that RP victims feel humiliated and have long-term disruptive mental health effects, hindering the well-being and quality of life of individuals. Humiliation is considered a response to social and interpersonal interactions, where psychological well-being and the fundamental sense of dignity (worth) are diminished or severely harmed by negatively affecting the person's affective-emotional organization (Hartling & Lindner, 2016; Leidner et al., 2012), which seems to be the case of RP victimization. Humiliation can result from a traumatic event where one person has exerted power over another, often leading to long-term consequences such as losing the ability to trust others (Leask, 2013). This definition of humiliation is in line with the negative consequences resulting from RP, with many victims reporting that their ex-partners exercised power over them at the time they disseminated their intimate content (Hall & Hearn, 2019), losing control of the situation, leading to a loss of trust in others (Bates, 2017). Therefore, the present study's results led to the belief that RP victims tend to reveal higher levels of humiliation because of their victimization, losing trust in others, as mentioned in the literature (Bond & Tyrrell, 2018). Other authors also argue that humiliation is present in various adverse events and is characterized by the negative focus given to individuals who experience adverse situations such as being harassed, ridiculed, and demeaned (Elison & Harter, 2007). RP victims usually report these situations (Aborisade, 2022; Bates, 2017). After victims have access to their intimate content on the internet, many of them feel so humiliated that they cannot cope with this feeling, making drastic changes in their lives in order to minimize the negative feelings, such as switching jobs, deleting their social media accounts, or even changing their residence so that they are not recognized and do not have to deal with public humiliation (Bates, 2017; Citron & Franks, 2014). Feelings of humiliation can respond to adverse events and are often associated with developing mental health complications, highlighting the importance of preventing these events from minimizing harm (Torres & Bergner, 2010).

### Limitations

Despite the important contributions, some limitations should be acknowledged. The first limitation of the study is the non-representativeness of the sample and the fact that it is composed exclusively of female individuals. Although the literature mentions that most victims of RP are female (Souza, 2020), this limitation does not allow generalizing the results to the Portuguese population. Thus, future studies should include larger, more heterogeneous samples of participants’ sex. The fact that the participants in the study had to be adults can also be considered a limitation because, according to the literature, adolescents who have not yet reached the age of majority are a particular risk group for RP victimization (Henry et al., 2017). In this sense, future research should consider younger groups, namely adolescents and young adults. Another limitation of the study is related to the online format of the survey, which made it impossible to control the environment when filling out the protocol, namely the control of social desirability. It is also essential to consider other variables in the future, such as self-blame, suicidal ideation, social isolation, change of residence of the victims, and change of personal identification of the victims, since previous studies mentioned these outcomes as frequent consequences of RP victimization (Franklin, 2014; Salter & Crofts, 2015; Wolak & Finkelhor, 2016). Including the variables in future studies will expand the knowledge about the phenomenon and develop and implement more effective prevention strategies and intervention plans suited to the victims’ needs.

## Conclusions

This research gives us important contributions and insights into the scientific and practical understanding of the RP phenomenon. First, this study highlights that RP is a relatively frequent phenomenon among young women associated with severe mental health consequences for the victims. Thus, it is helpful to develop structured prevention strategies, and intervention plans empirically informed to reduce online victimization and its negative impact. More restrictive laws and policies regarding sharing non-consented sexual content should also be developed and implemented. The study also contributes to a better understanding of the risks associated with using the Internet, making it worth developing training focused on the risks associated with the Internet, particularly in intimate relationships, to prevent the practices of RP and other similar forms of online victimization.

In sum, this study found that a considerable percentage of young women were victims of at least one act of RP lifetime and that victims of RP tend to have low self-esteem and high feelings of humiliation, depression, and anxiety. The present study can be relevant not only for understanding the phenomenon of RP and for in-depth knowledge of the inherent risks but also for developing prevention strategies and adequate intervention plans to diminish the possible consequences of this phenomenon.
